# A Novel Bifunctional Hybrid with Marine Bacterium Alkaline Phosphatase and Far Eastern Holothurian Mannan-Binding Lectin Activities

**DOI:** 10.1371/journal.pone.0112729

**Published:** 2014-11-14

**Authors:** Larissa Balabanova, Vasily Golotin, Svetlana Kovalchuk, Alexander Bulgakov, Galina Likhatskaya, Oksana Son, Valery Rasskazov

**Affiliations:** 1 Laboratory of Marine Biochemistry, G.B. Elyakov Pacific Institute of Bioorganic Chemistry, Far Eastern Branch, Russian Academy of Sciences, Vladivostok, Russian Federation; 2 School of Economics and Management, Far Eastern Federal University, Vladivostok, Russian Federation; University of Leicester, United Kingdom

## Abstract

A fusion between the genes encoding the marine bacterium *Cobetia marina* alkaline phosphatase (*Cm*AP) and Far Eastern holothurian *Apostichopus japonicus* mannan-binding C-type lectin (MBL-*AJ*) was performed. Expression of the fusion gene in *E. coli* cells resulted in yield of soluble recombinant chimeric protein *Cm*AP/MBL-*AJ* with the high alkaline phosphatase activity and specificity of the lectin MBL-*AJ*. The bifunctional hybrid *Cm*AP/MBL-*AJ* was produced as a dimer with the molecular mass of 200 kDa. The *Cm*AP/MBL-*AJ* dimer model showed the two-subunit lectin part that is associated with two molecules of alkaline phosphatase functioning independently from each other. The highly active *Cm*AP label genetically linked to MBL-*AJ* has advantaged the lectin-binding assay in its sensitivity and time. The double substitution A156N/F159K in the lectin domain of *Cm*AP/MBL-*AJ* has enhanced its lectin activity by 25±5%. The bifunctional hybrid holothurian's lectin could be promising tool for developing non-invasive methods for biological markers assessment, particularly for improving the MBL-*AJ*-based method for early detection of a malignant condition in cervical specimens.

## Introduction

The peculiar characteristic to recognize and bind specific carbohydrates made lectins of animal and plant origin useful tools for detecting changes in carbohydrate profiles and identifying aberrant glycans in neoplastic cells with the aim of more precise diagnostics and more accurate prognosis. The technique most common and widespread is the use of lectins in immunohistochemical assays [1], [2], [3], [4]. Molecules with a narrow specificity, which are able to bind selectively to carbohydrates, have also a key importance in the development of research related with mechanism of cancerogenesis or inflammation at the molecular level as well as for designing drugs targeted to a relevant molecule. In consequence of the strong innate defense system and the absence of the adaptive immunity in marine invertebrates, a number of their lectins were found to play considerable biological recognition role and therefore have unique specific activities. Several methods regarding the use of marine invertebrate lectins, including mannan-binding C-type lectin from Far Eastern holothurian MBL-*AJ*, as tools for recognizing aberrant glycans or foreign microbial structures have been proposed in recent days [5], [6], [7], [8], [9], [10], [11], [12]. In addition, MBL-*AJ* was successfully applied for differential diagnostics of benign and malignant neoplasms of uterine cervix by the analysis of contents of lectin-binding carcinoembryonic antigen (CEA) in vaginal secretion [13]. The native MBL-*AJ* has oligomeric forms with identical 17-kDa subunits and natural ligands among extracellular low-branched bacterial α-D-mannans composed of α-1,2- and α-1,6-linked D-mannose residues, participating in recognition of the altered structures in the invertebrate during embryogenesis, morphogenesis and the formation of the immune response [5], [10], [11]. Cross-reactivity of MBL-*AJ* and human serum lectin MBL detected by the antibodies against MBL-*AJ* suggested the presence of common antigenic determinants [5]. However, the MBL-*AJ* specificities resulted in the absence of the MBL-*AJ* interaction with the components of the healthy patient's serum have been found to facilitate the detection of the slight structural differences of glycans, excluding the wrong positive results in the assayed samples [13]. Although cancers of ovaries, cervix and uterus are regarded as difficult and expensive for the detection at an early stage [13], [14], [15], [16], the method with the use of MBL-*AJ* has allowed identifying statistically reliable differences between the levels of the lectin-binding CEA between healthy women and patients with cervical cancer, and between patients with benign and malignant neoplasm [13]. Moreover, it is important that MBL-*AJ*-based method gives a possibility to assess tumours noninvasively.

To improve the MBL-*AJ*-binding assay for detection of aberrant patterns of glycosylation a novel bifunctional hybrid protein *Cm*AP/MBL-*AJ* based on the Far Eastern holothurian lectin MBL-*AJ* gene fused with the gene of the marine bacterium alkaline phosphatase *Cm*AP has been synthesized with *E. coli* cells. *Cm*AP/MBL-*AJ* has the binding activity of lectin improved by mutations and the high enzymatic activity of alkaline phosphatase facilitating the identification of MBL-*AJ* ligands without the additional steps of the MBL-*AJ*-specific antibodies production and conjugation with an enzyme.

## Materials and Methods

### Construction of recombinant plasmids

Expression plasmid encoding the fusion protein *Cm*AP/MBL-*AJ* has been constructed on the base of NcoI/SalI part of pET-40b(+) vector (Novagen) and the chimeric gene comprised the mature *Cm*AP open reading frame (accession n. DQ435608) and cDNA fragment encoding mature lectin MBL-*AJ* (accession n. AAT42221) linked by the sequence encoding the peptide linker (G_4_S)_3_. For the *Cm*AP gene amplification, marine bacterium *Cobetia marina* KMM 296 (Collection of Marine Microorganisms PIBOC FEB RAS) chromosomal DNA, Encyclo Taq Polymerase (Evrogen), and the gene-specific upstream primer, 5′– TATTCCATGGCAGAGATCAAGAATGTCATTCTGAT-3′, and downstream primer including the linker (G_4_S)_3_, 5′ – TTAAGAGCTCAGAACCACCACCACCAGAACCACCACCACCAGAACCACCACCACCCTTCGCTACCACTGTCTTCAGATACTGTCCT – 3′, were used. The recombinant plasmid pET40*Cm*AP was obtained by ligation of *Cm*AP gene into pET-40b(+) and used for the subsequent cloning of the lectin MBL-*AJ* gene.

For the lectin gene amplification, cDNA of *A. japonicus* coelomocytes, the gene-specific upstream primer, Lect-X-dir: 5′– AGCTGAGCTCTGTCTGACGGCTTGTCCGGAGTTTTG– 3′, and downstream primer, Lect-X-rev: 5′– CAGTGTCGACCTCCAAATGATACTCGATACAGGGAAG-3′ (construction 1 –*Cm*AP/MBL-*AJ*-Stop_codon), or X-Lect-pET40rev-TEV: 5′– ACTGGTCGACTTAGTGGTGGTGGTGGTGGTGGTGGTGACCCTGAAAATAAAGATTCTCCAAATGATACTCGATACAGGGAAG– 3′ (construction 2 – *Cm*AP/MBL-*AJ*-TEV) were used. cDNA for MBL-AJ was produced as previously described [5]. The plasmid pET40*Cm*AP/MBL-*AJ* was obtained by ligating the cDNA fragment encoding MBL-*AJ* into the recombinant vector pET40*Cm*AP, linearized by SacI/SalI site-specific scission. Restriction endonucleases and T4 DNA ligase were purchased from Fermentas. The PCR product and linearized plasmid were purified from agarose gel with Gel Extraction kit (Qiagen). Correct recombinant plasmid sequences were verified by DNA sequencing using an automated PE/ABI 310 DNA sequencer and PE/ABI-ABI PRISM BigDye Terminator cycle sequencing Ready Reaction Kit (PE Applied Biosystems). Preparation of *E.coli* DH5α competent cells for plasmid propagation and heat shock transformation were carried out according to the standard method [17]. All biochemicals and reagents were from Fermentas and Sigma-Aldrich. *E. coli* Rosetta (DE3) cells were used as a host for the expression of the recombinant hybrid *Cm*AP/MBL-*AJ*.

### Expression of the recombinant hybrid *Cm*AP/MBL-*AJ*

For the expression of *Cm*AP/MBL-*AJ*, the *E.coli* Rosetta(DE3) cells transformed with the recombinant plasmid pET40*Cm*AP/MBL-*AJ* were grown on LB agar plate containing 25 mg/ml kanamycin overnight at 37°C. A single colony was picked and grown at 200 prm in 20 mL of LB medium with 25 mg/ml kanamycin at 37°C for 12 h, then transferred to 1 L of fresh LB with 25 mg/ml kanamycin. When the cell density reached an A_600_ of 0.6–0.8, 0.2 mM IPTG was added to induce the expression of the protein, and the incubation continued at 16°C up to 12 h at 200 rpm. *E.coli* Rosetta(DE3) cells were transformed with pET-40b (+) as a control.

### The recombinant hybrid *Cm*AP/MBL-*AJ* purification

All purification steps were carried out at +6°C. After harvesting, the *E.coli* cells were resuspended in 150 ml of the buffer A, containing 0.02 M Tris-HCl, pH 8.2, and 0.01% NaN_3_, and disintegrated by ultrasonic treatment, then centrifuged at 10000 g for 30 min. The supernatant was applied to a column (4×25 cm) of DEAE-52 cellulose (Whatman). Elution of the protein was performed with NaCl gradient (0.05 M–0.38 M) in the buffer A. The enzymatically active fractions were collected and applied to a column (1×2.5 cm) of Ni-agarose (Qiagen). Elution of the protein was carried out in buffer B (0.02 M Tris-HCl, pH 8.2, 0.05 M EDTA, 0.01% NaN_3_). The active fractions were collected and desalted with DEAE-Toyopearl 650 M (Toyo Soda) column (0.7×2.5 cm), then incubated with TEV protease (New England Biolabs) at 21°C for 12 hours. Then the protein solution was applied to a gel filtration column (1.5×170 cm) of Superdex 200 (Sigma) equilibrated with buffer C (0.02 M Tris-HCl, pH 8.2, 0.1 M NaCl, 0.01% NaN_3_). All purification steps were evaluated by SDS-PAGE according to Laemmli [18]. The concentration of the protein was determined according to Bradford [19]. Peptide sequencing of the N-terminal end was performed on Edman Automated Sequencing Apparatus Beckman 890C.

### Enzymatic activity assay

The standard assay for alkaline phosphatase (AP) activity was carried out at 37°C using 2 mM *p*-nitrophenylphosphate (*p*NPP) (Sigma Chemical Co.) in 0.1 M Tris-HCl buffer, pH 9.0, containing 0.2 M KCl, or in 1 M diethanolamine (DEA) buffer, pH 10.3, containing 15–20 mM *p*NPP. The release of ***p***-nitrophenol (***ε*** = 18.5 mM^−1^·cm^−1^) was monitored at 405 nm. One unit of AP activity was defined as the quantity of the enzyme required to release 1.0 µmol of *p*-nitrophenol from *p*NPP in 1 min. The specific activity was calculated as units per 1 mg of protein.

### Lectin activity assay

The embryonic alpha-1-acid glycoprotein (0.1 µg/mL) was dissolved in 0.1 M carbonate buffer, pH 9.5, containing 0.15 M NaCl, and 150 µl was added to the first well and was two-fold serially diluted in the same buffer in each well of a polystyrene 96-well ELISA microtiter plate (Maxisorp, Nunc), and then incubated at 4°C overnight. After incubation, the plate was washed three times with the buffer containing 0.01 M Tris-HCl, pH 7.5, 0.15 M NaCl, 0.05% Triton X-100 (TBS-T) and then three times with water. After that, 160 µL of bovine serum albumin (1 µg/mL) in TBS-T was added to each well to block non-specific binding sites. The plate was then incubated for 1 hour at room temperature and washed as described above. 150-µL aliquots of the samples containing chimeric lectin *Cm*AP/MBL-*AJ* with concentration 100 µg/mL were added to each well. TBS-T was used as negative control, and interaction of MBL-*AJ* with adsorbed antibodies under the same conditions served as a positive control. After that, the plate was kept for 1 h at room temperature and washed again. Then 150 µL of *p*NPP in the buffer for AP assay was added to each well. After 2–5 min of incubation, 100 µL of 2 M sodium hydroxide was added to stop the reaction. Absorbance of the resultant solution was measured at 400 nm with a plate spectrophotometer (Bio-Tek Instruments, USA).

### Molecular mass determination

The molecular size of the native hybrid *Cm*AP/MBL-*AJ* was determined by gel filtration on the column (1.5×170 cm) of Superdex 200 (Sigma) in 20 mM Tris-HCl, pH 8.2, 100 mM NaCl at a flow of 0.2 ml/min at 6°C and calibrated using Bio-Rad standard molecular weight markers: bovine thyroglobulin (670 kDa), bovine γ-globulin (158 kDa), chicken ovalbumin (44 kDa), horse myoglobin (17 kDa), Vitamin B_12_ (1.35 kDa). The molecular mass of *Cm*AP/MBL-*AJ* was determined by 12.5% SDS polyacrylamide gel electrophoresis (SDS-PAGE). An aliquot of the protein solution was mixed with Laemmli sample buffer and heated at 95°C for 5 min, then applied to SDS-PAGE and stained according to Laemmli [18]. PAGE for the native *Cm*AP/MBL-*AJ* was performed without SDS and heat treatment. The “native” gels were stained for AP by the casing over with the standard enzyme assay buffer containing 2 mM *p*NPP. Both PAGEs were performed at 25°C.

### Molecular modeling

The structural models of *Cm*AP and MBL-*AJ* were generated by comparative modeling approach for further modeling the putative spatial structure of the bifunctional hybrid *Cm*AP/MBL-*AJ*. The target-template alignments and modeling of *Cm*AP and MBL-*AJ* 3D-structures were carried out by the molecular modeling package Molecular Operating Environment version 2013.08 (Chemical Computing Group Inc., 1010 Sherbooke St. West, Suite #910, Montreal, QC, Canada, H3A 2R7, 2013). The high-quality experimental structure of alkaline phosphatase from marine bacterium *Vibrio* sp. G-15 with 1.4 Å resolution (VAP; PDB code: 3E2D) was used as a template for homology modeling of *Cm*AP 3D-structure. The crystal structure of C-type lectin CEL-IV from holothurian *Cucumaria echinata* with 1.65 Å resolution (PDB code: 3ALU) was used as a template for homology modeling of MBL-*AJ* 3D-structure. The *Cm*AP/MBL-*AJ* chimeric model was manually generated by adding (G_4_S)_3_ linker to the C-terminus of the *Cm*AP model and by binding with N-terminus of MBL-*AJ* using program MOE. Structural optimization of *Cm*AP, MBL-*AJ* and *Cm*AP/MBL-*AJ* models were carried out by energy minimization method with force field Amber12:EHT using program MOE 2013.08. The structure of the model oligosaccharide α-D-Manp-(1→6)-[α-D-Manp-(1→2)-α-D-Manp-(1→2)]-α-D-Manp(1→6)-D-Manp was build and optimized in water with MOE. The single W100A, Q103H, A156N, F159K, double A156N/F159K MBL-*AJ* mutants were *in silico* generated with program MOE. The molecular docking of the MBL-*AJ* mutants with the model branched oligosaccharide α-D-Manp-(1→6)-[α-D-Manp-(1→2)-α-D-Manp-(1→2)]-α-D-Manp(1→6)-D-Manp was carried out for the calculation of binding energy and affinity values [20] to design the lectin configuration with a highest affinity to branched oligosaccharide. The binding energy or solvation (the molecular mechanics generalized Born interaction energy) was the non-bonded interaction energy between the receptor and the ligand and comprised van der Waals, Coulomb and generalized Born implicit solvent interaction energies [20]. The affinity (the estimated binding affinity) was that of the GBVI/WSA dG scoring function reported in units of kcal/mol [20]. The evaluation of structural parameters, analysis of protein-ligand contacts, physico-chemical properties, molecular docking and visualization of the results were carried out with the modules of MOE 2013.08 program.

### Site-specific mutagenesis of lectin-binding domain of *Cm*AP/MBL-*AJ*

The single W100A, Q103H, A156N, F159K and double A156N/F159K mutants of *Cm*AP/MBL-*AJ* were genetically engineered by site-directed mutagenesis. The single or double amino acid substitutions were introduced into the forward and reverse gene-specific primers to the expected gene regions. The resultant PCR products were purified from agarose gel and annealed both the DNA matrix and primers in the equivalent ratio for the next PCR. The resultant mutant gene was amplified with the primers Lect-X-dir and Lect-X-rev, and fused to the linearized vector pET40*Cm*AP and expressed as described above.

The lectin-binding activity assay of the mutant *Cm*AP/MBL-*AJ* colonies was carried out by the modified method described above. Mucin (0.1 µg/mL) was dissolved in 0.1 M carbonate buffer, pH 9.5, containing 0.15 M NaCl, and 150 µl was added to each well of a polystyrene 96 -well ELISA microtiter plate (Maxisorp, Nunc), and then incubated at 4°C overnight. After incubation, the plate was washed three times with the buffer, containing 0.01 M Tris-HCl, pH 7.5, 0.15 M NaCl, 0.05% Triton X-100 (TBS-T) and then three times with water. After that, bovine serum albumin (1 µg/mL) in TBS-T was added as described above. Samples containing chimeric lectin *Cm*AP/MBL-*AJ* (100 µg/mL) were two-fold serially diluted in TBS-T and added in 150 µL aliquots to each well. TBS-T was used as a negative control. After that, the plate was incubated, washed and visualized as described above. All of the lectin activity assays were performed in 3 independent parallels for 3 to 5 times.

### Statistics

All values presented in this article are representative of at least three independent experiments. Data were analyzed using the Student's t-test of the SigmaPlot 2000 version 6.0 program (SPSS Inc.). Differences from controls were considered significant at P≤0.05.

## Results

### Expression and purification of the bifunctional hybrid *Cm*AP/MBL-*AJ*

A novel method for producing a highly carbohydrate-specific holothurian lectin MBL-*AJ* has been developed. The recombinant bifunctional hybrid *Cm*AP/MBL-*AJ* expression and purification were monitored by the alkaline phosphatase *Cm*AP activity facilitating the rapid and effective detection of the *Cm*AP/MBL-*AJ*-containing fractions. Although the expression of all engineered the full-length mature *Cm*AP and MBL-*AJ* genes fusions in *E. coli* cells resulted in a high-level production of AP activity, the construction (2) containing C-terminal 6XHis tags was more useful for the hybrid *Cm*AP/MBL-*AJ* purification than the same construction (1) without C-terminal 6XHis tag. The additional His-tag drastically enhanced the binding of *Cm*AP/MBL-*AJ* to Ni-agarose column. However, for increasing of the lectin-binding activity of *Cm*AP/MBL-*AJ*, the step of the TEV protease cleavage of C-terminal 6XHis tag was preferable (Table 1).

**Table 1:** Purification of bifunctional hybrid *Cm*AP/MBL-*AJ*.

Purification stage	Total protein, mg	Specific activity (Tris-HCl/DEA, units/mg	Yield, %
Crude cell extract	206	12.6/42	100
DEAE-52 Cellulose	67	52/177	63
Ni-NTA agarose	6.2	464/1578	51
*TEV protease cleavage (*optionally for construction (2))	6.2	452/1537	50
Superdex 200	1.1	1576/5100	24

**Figure 1:**
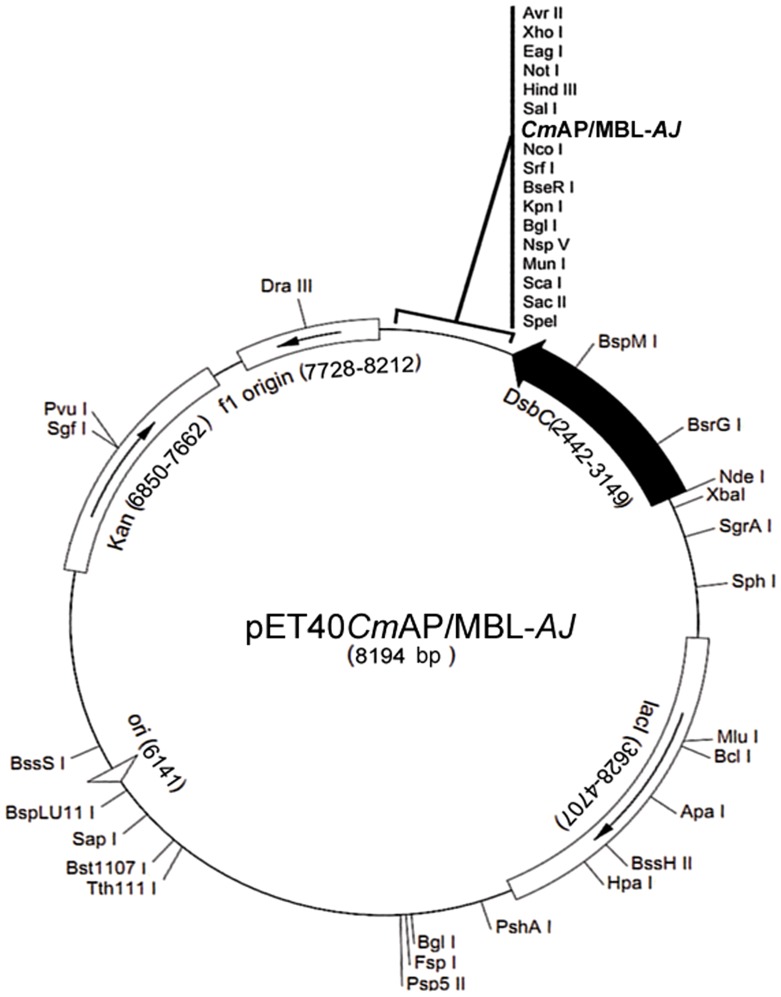
Scheme of expression plasmid pET40CmAP/MBL-AJ.

As a result, the *Cm*AP/MBL-*AJ* production reached up to 1.1 mg of the final yield of the functionally active protein from 1 L of the *E.coli* Rosetta(DE3) cells culture carrying the expression recombinant vector pET40*Cm*AP/MBL-*AJ* (Table 1, Fig. 1).

**Figure 2:**
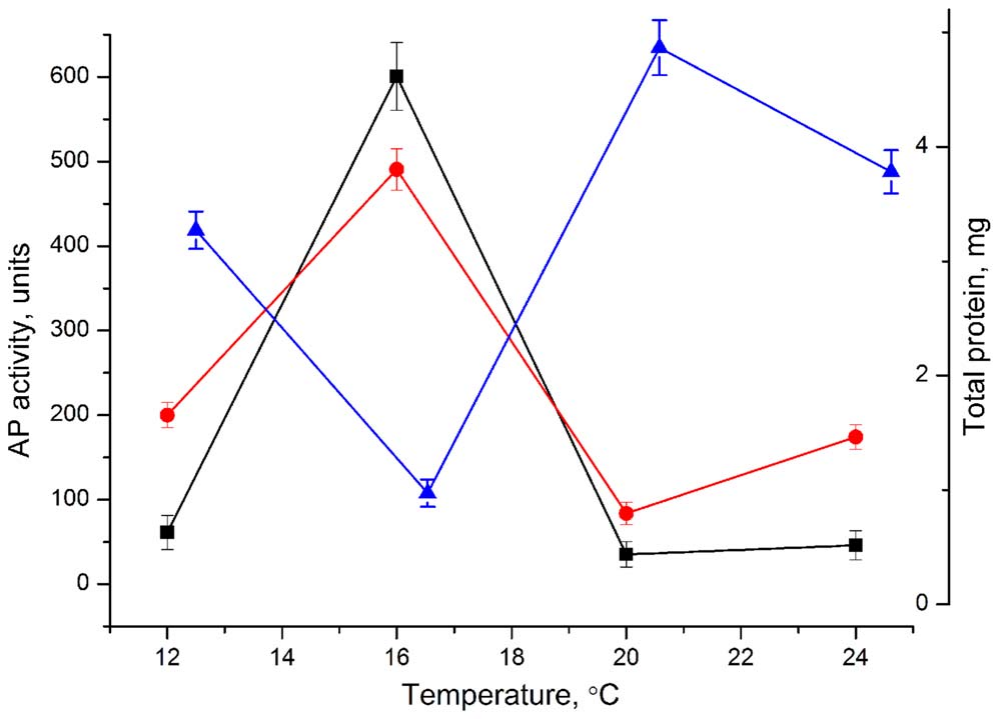
Optimization of pET40CmAP/MBL-AJ expression: black squares - specific AP activity; red circles - total activity; blue triangles - total protein.

**Figure 3:**
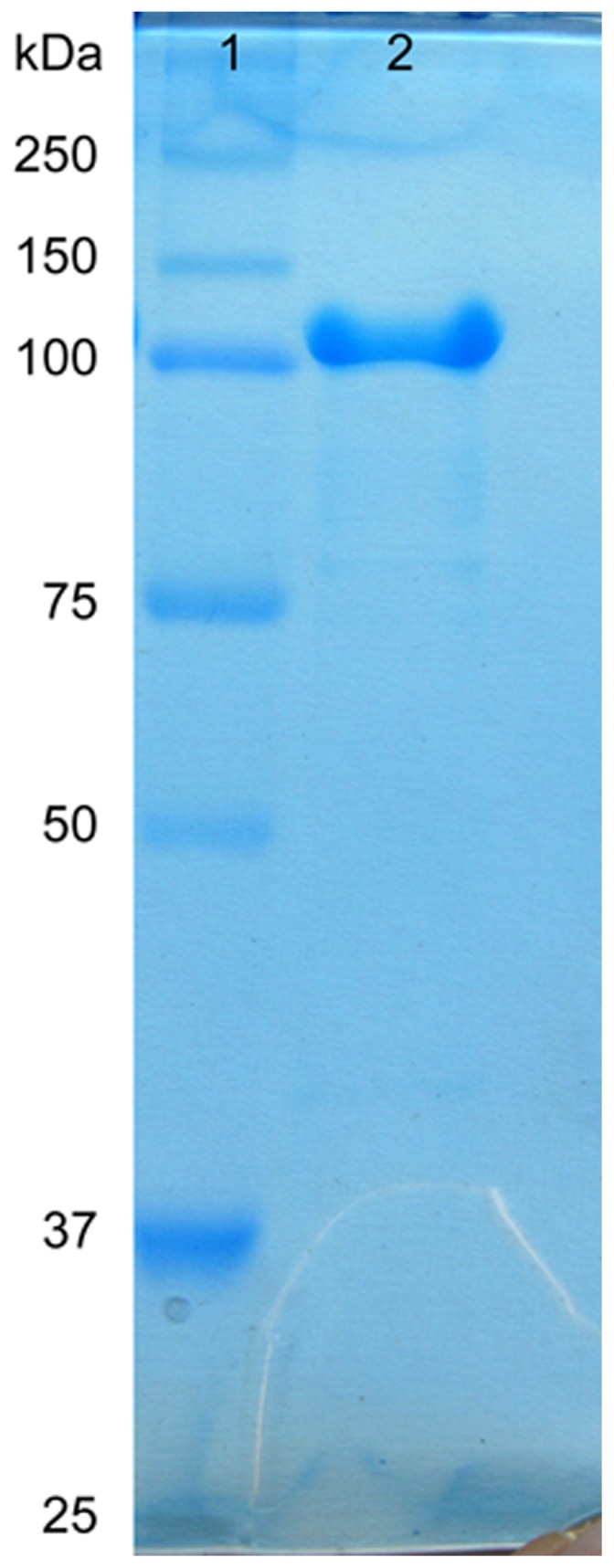
SDS-PAGE of *Cm*AP/MBL-*AJ* eluted from Superdex 200: 1 –molecular weight marker (Bio-rad); 2 –*Cm*AP/MBL-*AJ*.

The optimal conditions for the *Cm*AP/MBL-*AJ* expression were the 0.2 mM concentration of IPTG and the strain cultivation at 16°C for 12 h (Fig. 2). At the final step of purification, SDS-PAGE showed a single band of the protein with a molecular mass of approximately 100 kDa, corresponding to the calculated molecular mass of the hybrid protein *Cm*AP/MBL-*AJ* consisted of the mature proteins of *Cm*AP (55 kDa), MBL-*AJ* (17 kDa), peptide linker (G_4_S)_3_, the plasmid fusion protein DbsC (32.5 kDa) and N- and C-terminal 6 X His tags (Fig. 1, 3). The relative molecular size determined by gel filtration corresponded to 200 kDa, suggesting a homodimeric form of the enzymatically active hybrid *Cm*AP/MBL-*AJ. Cm*AP/MBL-*AJ* appeared as tetramer at the highly alkaline pH≥9 (data not shown).

### Enzymatic and lectin properties of the bifunctional hybrid *Cm*AP/MBL-*AJ*

**Figure 4:**
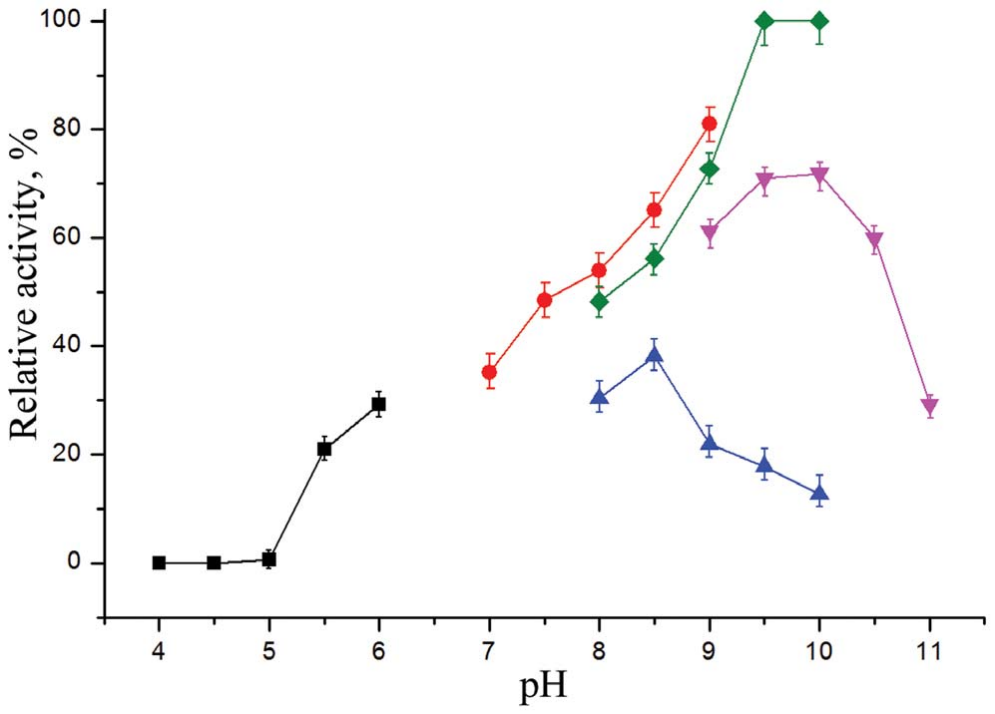
Stability of *Cm*AP in different buffers. The enzyme activity was measured using the standard enzyme assay (0.1 M Tris-HCl buffer, pH 9.0) after 5 h incubation at different pH values. Squares – 0.03 M Na COOH_3_ (4.0–6.0); circles - 0.05 M Tris-HCl (7.0–9.0); diamonds – 1 M DEA (8.0–10.3); down triangle - 0.1 M NaHCO_3_ (9.0–11.0); up triangle - 0.1 M glycine (9.0–11.0) (n = 9, p≤0.05).

The recombinant bifunctional hybrid *Cm*AP/MBL-*AJ* has an optimal AP activity at pH 9.0–10.3 in 1 M DEA or 0.1 M Tris-HCl buffer containing 0.2 M KCl or NaCl, and does not depend on the presence of the bivalent metal ions that accords with the properties of wild type of the enzyme (Plisova et al. 2005). *Cm*AP/MBL-*AJ* is stable at pH between 6.0–11.0, but *Cm*AP is gradually inactivated below pH 6.0. *Cm*AP was also gradually inhibited when the enzyme was exposed above pH 8.5 maintained by 0.1 M glycine (Fig. 4).

*Cm*AP/MBL-*AJ* has temperature optimums 40°C–50°C for the manifestation of the highest AP activity of 5100 U/mg in 1 M DEA buffer or 1500 U/mg in 0.1 M Tris-HCl buffer, 0.2 M KCl, at pH 10.0 (Table 1). *Cm*AP/MBL-*AJ* has a half-life of 27 min at 40°C and 16 min at 45°C in the presence of 2 mM EDTA (data not shown). Inactivation of 50% of AP activity occurred in 45 min at 37°C. However, *Cm*AP/MBL-*AJ* was stable up to 45°C with a half-life of 15 min at 47°C and 120 min at 45°C, when the enzyme was incubated with 10 mM MgCl_2_ (data not shown).

The level of lectin-binding activity of the bifunctional hybrid *Cm*AP/MBL-*AJ* was estimated by its affinity to embryonic alpha-1-acid glycoprotein or mucin. The detailed carbohydrate-binding specificity of MBL-*AJ* determined by inhibition assay has been previously shown [5].

**Figure 5:**
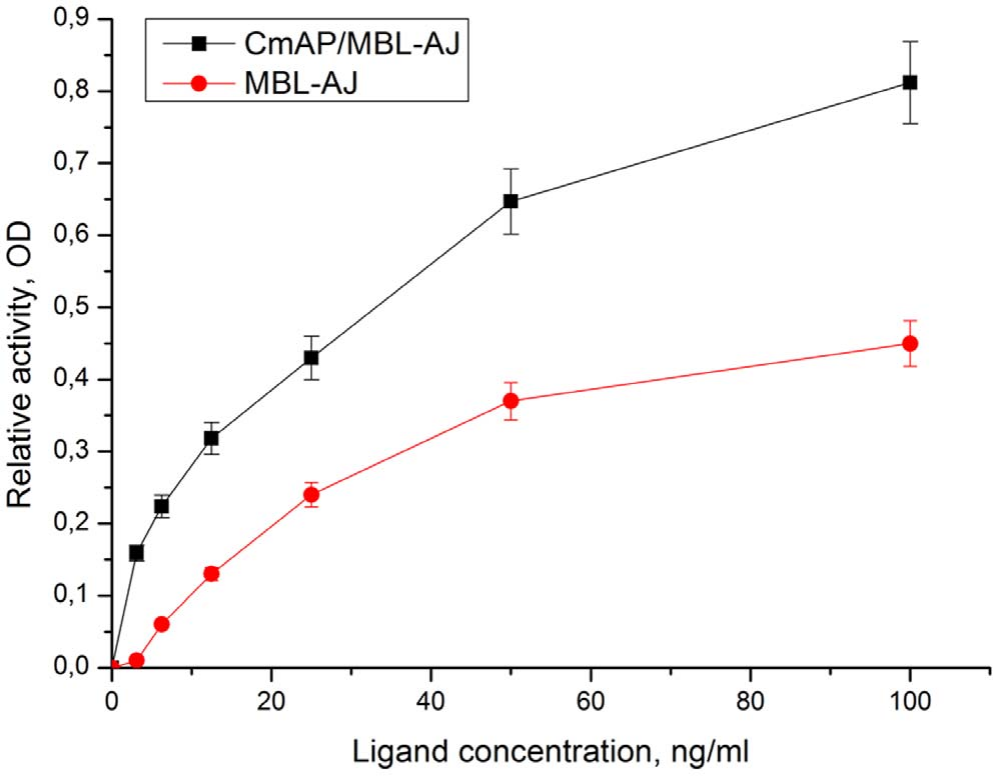
Determination of MBL-AJ-binding embryonic alpha-1-acid glycoproteins by the two methods: with the use of hybrid CmAP/MBL-AJ (black) and MBL-AJ-antibody-horse peroxidase conjugates (red).

The highly active AP label genetically linked to the lectin MBL-*AJ* has advantaged the lectin-binding assay in its sensitivity and time in comparison with the two-step method of the wild-type MBL-*AJ* conjugation with the lowly active horse peroxidase (Fig. 5).

The lectin activity of the bifunctional hybrid *Cm*AP/MBL-*AJ* is Ca^2+^ and pH-dependent and reversibly inhibited by EDTA. The optimum buffer for the lectin activity of *Cm*AP/MBL-*AJ* is 0.1 M Tris-HCl, 0.15 M NaCl, 0.01 M CaCl_2_, pH 7.0–9.0 that is suitable for both the AP and lectin activities. The highest affinity of *Cm*AP/MBL-*AJ* to ligands is observed at 4°C. However, *Cm*AP can also exhibit an appreciable hydrolytic activity at the low temperature due to its cold-adaptability (data not shown). The diluted of the highly purified *Cm*AP/MBL-*AJ* preparation up to thousand fold was fully stable at −20°C during more than one-year storage in the dilution buffer (0.02 M Tris-HCl, pH 8.2, 0.01% NaNO_3_).

### Molecular modeling of the bifunctional hybrid *Cm*AP/MBL-*AJ*

Search for a template of *Cm*AP showed that the crystal structure of the cold-active *Vibrio* sp. G15-21 alkaline phosphatase (VAP; PDB ID 3E2D) obtained with 1.4 Å resolution [21] and the crystal structure of alkaline phosphatase from the moderate halophilic bacterium *Halomonas* sp. 593 (HaALP) determined with 2.1 Å resolution recently [22] can be used to generate a homology model.

**Figure 6:**
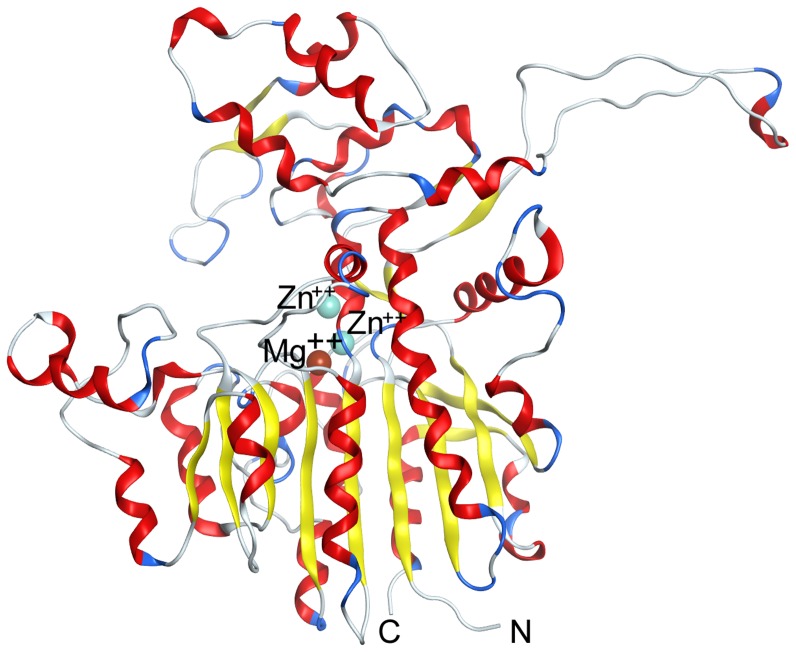
The homology model of *Cm*AP monomer with binding sites for two Zn^2+^ and one Mg^2+^ ions.

Amino acid sequences of *Cm*AP, HaALLP and VAP have 75% and 69% of identity and 86% and 82% of similarity, respectively. The 3D-structure of cold-active alkaline phosphatase *Cm*AP from marine bacterium *C. marina* (Uniprot Q1W622) was modeled using as a template the crystal structure of the cold-active *Vibrio* sp. G15-21 alkaline phosphatase (Fig. 6).

Superposition of all Cα atoms of *Cm*AP model and template showed that the value of root mean square deviation (RMSD) is 0.43 Å. The structure of the *Cm*AP monomer is stabilized by 148 hydrogen bonds, 20 ionic bonds and 180 hydrophobic contacts. The disulfide bonds are absent in *Cm*AP, HaALP and VAP structures. Superposition of the *Cm*AP structural model and HaALP and VAP crystal structures showed that the value RMSD is 0.87 Å for 497 Cα atoms. The *Cm*AP monomer structure consists of a catalytic domain and a “crown”-domain by analogy with VAP and HaALP. Catalytic domain displays a three-layer β-sandwich fold and contains β-sheet of nine β-strands surrounded by α-helices at both sides. The structure of the catalytic domain has a fold of (α-β-α)-type that is AP characteristic. The *Cm*AP, HaALP and VAP “crown”-domains are the largest observed in known APs. The cold-active *Cm*AP, HaALP and VAP are the AP variants with the highest known k(cat) value. Analysis of the *Cm*AP 3D-structure models and crystal structures of VAP and HaALP showed that the structures of the active sites and metal binding sites of *Cm*AP, HaALP and VAP are identical. The critical N-terminal region for the stability of dimeric ECAP from *E. coli* K12 is absent in *Cm*AP as in VAP and HaALP. The VAP or HaALP crystal structures are the dimers but it was experimentally showed that the functional structure of the *Cm*AP is a monomer [23].

**Figure 7:**
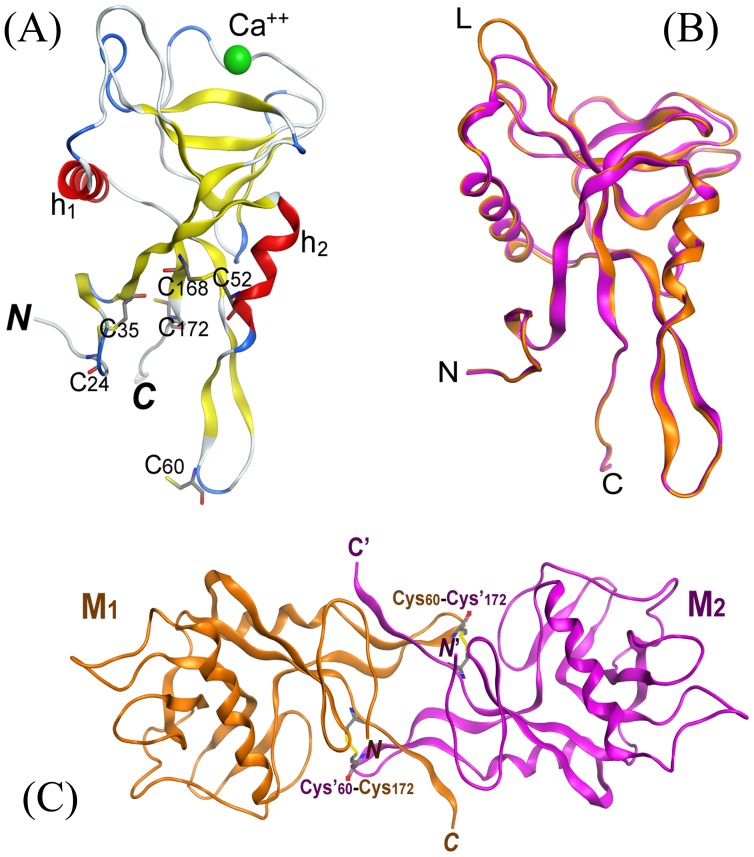
The homology model of MBL-*AJ* monomer (A), the superimposition of MBL-*AJ* model with template CEL-IV (B) and the structural model of MBL-*AJ* dimer (C). (a) - Graphic representation of MBL-*AJ* monomer with disulfide bonds (Cys24-Cys35, Cys52-Cys168) and free Cys60 and Cys172 group at C-terminus of lectin displayed as sticks. (b) - Superimposition of the MBL-*AJ* structural model (orange) and template CEL-IV (purple). Loop L of MBL-*AJ* (orange) with significant difference is indicated. (c) – The structural model of MBL-*AJ* dimer build from monomer M1 (orange) and M2 (purple) with two intermonomer disulfide bonds Cys′60-Cys172 and Cys60-Cys′172.

Structural model of lectin MBL-*AJ* was generated using as a template of the crystal structure of sea cucumber lectin CEL-IV (PDB ID 3ALU) (Fig. 7 (A)) [24].

**Figure 8:**
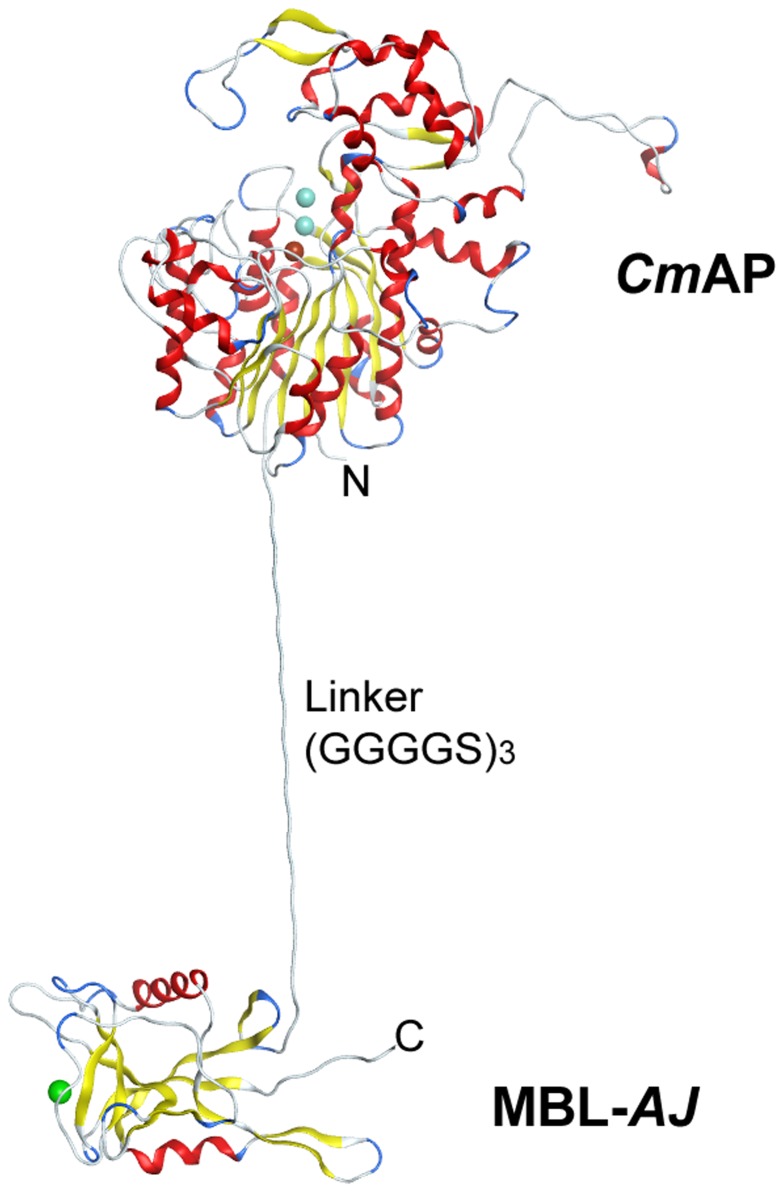
The structural model of the hybrid *Cm*AP/MBL-*AJ* monomer. The C-terminal region of *Cm*AP is linked via a linker (G_4_S)_3_ with the N-terminal region of MBL-*AJ*. Blue spheres indicate two ions Zn^2+^, red sphere indicates ion Mg^2+^ in *Cm*AP; green sphere indicates ion Ca^2+^ in MBL-*AJ*.

Amino acid sequence analysis of the lectin MBL-*AJ* and the template CEL-IV revealed that they have 65% identity and 79% similarity. Spatial structure of the lectin MBL-*AJ* is characteristic of the packed C-type lectins and contains two α-helix and 12 β-strands, disordered loops and ion Ca^2+^ in carbohydrate-recognizing domain. 3D-superposition of Cα- atoms of the structural model and prototype showed the value of RMSD to be 0.4 Å. The lectin MBL-*AJ* has a longer loop near the carbohydrate-recognizing domain than CEL-IV lectin (Fig. 7 (B)). The lectins MBL-*AJ* and CEL-IV have conservative position of six cystein residues that form two disulfide bonds stabilizing the monomer structure (Fig. 7 (A)). MBL-*AJ* dimer and tetramer are the functionally active forms of the lectin [5]. Therefore, the MBL-*AJ* dimer structure is generated using the crystal structure of the CEL-IV lectin dimer (Fig. 7 (C)). For the lectin CEL-IV it has been shown that two cysteine residues are involved in the formation of S-S bonds between monomers in the dimeric form of the lectin. Similar disulfide bond stabilizes the dimer structure of MBL-*AJ* (Fig. 7 (C)). The structure of the bifunctional hybrid *Cm*AP/MBL-*AJ* monomer consisting of the alkaline phosphatase *Cm*AP, linker (G_4_S)_3_ and lectin MBL-*AJ* was generated using MOE 2013.08 (Fig. 8).

**Figure 9:**
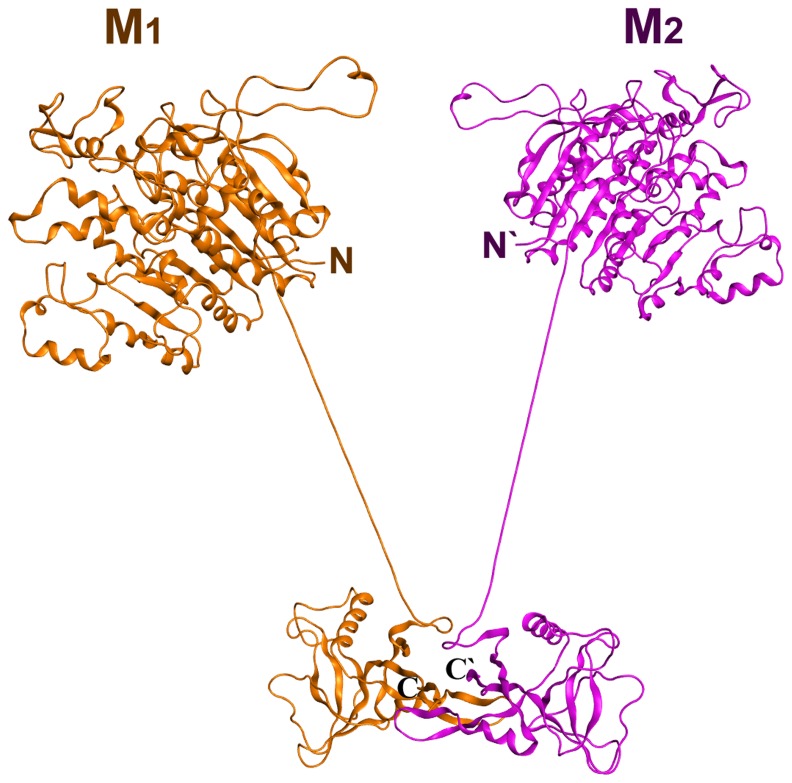
The putative structural model of hybrid *Cm*AP/MBL-*AJ* dimer built from monomers M1 (orange) and M2 (purple) on the base of the model MBL-*AJ* dimer.

**Figure 10:**
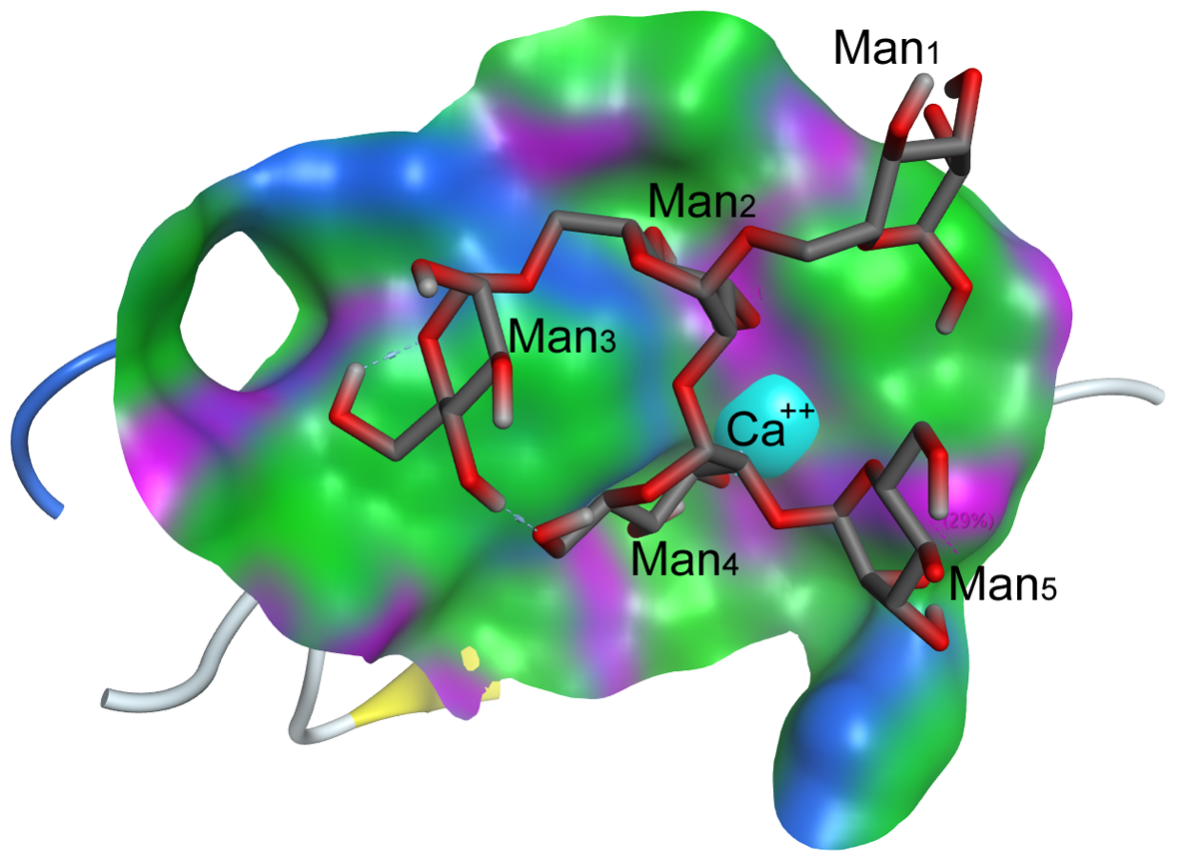
*In silico* docking the model of oligosaccharide α-D-Manp-(1→6)-[α-D-Manp-(1→2)-α-D-Manp-(1→2)]-α-D-Manp(1→6)-D-Manp inside of the MBL-*AJ* carbohydrate-recognition domain. Surface representation of the MBL-*AJ* binding site hydrophobic regions are in green, mildly polar regions are in blue and hydrogen bonding are in purple. Oligosaccharide structure is shown in the stick form, Ca^2+^ ion is shown as space filling.

**Figure 11:**
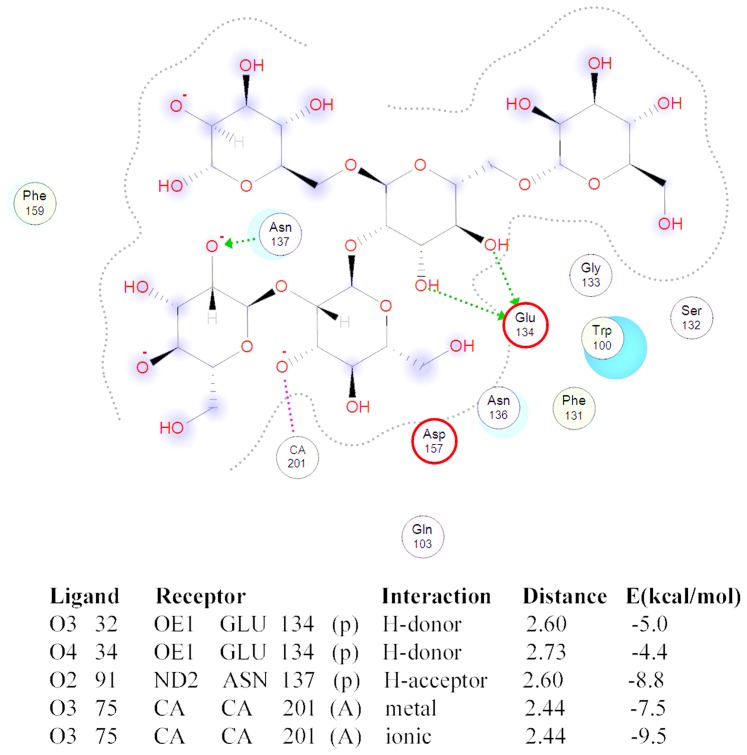
2D-Diagram of the oligosaccharide binding with MBL-*AJ* and contacts of the oligosaccharide with MBL-*AJ*.

Since the lectin MBL-*AJ* is a functionally active dimer, the hybrid *Cm*AP/MBL-*AJ* model was constructed based on the MBL-*AJ* dimer. The *Cm*AP/MBL-*AJ* dimer model has the two-subunit lectin part that is associated with two molecules of alkaline phosphatase functioning independently from each other (Fig. 9). The MBL-*AJ* lectin exhibits high specificity for branched mannan [5]. Molecular docking predicted the structural model of MBL-AJ lectin binding with a model oligosaccharide (Fig. 10). The mannose residues of the main chain Man2 and from branching chain Man4 that are not the terminal mannose residues form contacts with the MBL-*AJ* carbohydrate-recognition domain. Analysis of the contacts in MBL-*AJ* carbohydrate-recognition domain showed that the model oligosaccharide forms hydrogen bonds with the lectin and Ca^2+^ ion (Fig. 11). According to the lectin-oligosaccharide complex model, the residue Glu134 forms hydrogen bonds with O3 and O4 of the mannose residue Man2 from the main chain; Ca^2+^ ion forms ionic and metal bonds with O3 of the mannose residue Man4; residue Asn137 forms hydrogen bond with O2 of the mannose residue Man5.

### *In silico* mutation and site-specific mutagenesis of the lectin-binding domain of the bifunctional hybrid *Cm*AP/MBL-*AJ*

*In silico* design of the MBL-*AJ* lectin with an improved affinity and activity was carried out using point mutations and molecular docking approach. A high quality of the MBL-*AJ* model allowed estimating the role of its essential amino acid residues for the interaction of MBL-*AJ* with ligands by *in silico* mutation. The 3D-models of mutants W100A, Q103H, A156N, F159K, A156N/F159K were generated. Molecular dockings of mutants with a model oligosaccharide were carried out and oligosaccharide binding energies with mutants and affinities were predicted (Table 2). The models of W100A, Q103H, A156N and F159K mutants of the *Cm*AP/MBL-*AJ* lectin module were built to study the molecular basis of its lectin-binding interaction with the model branched oligomannan. The residues W100 and Q103 were selected for substitutions by the reason of their homology with the template CEL-IV residues W79 and Q82 participating in the binding of the mannose residues [24]. The residue substitution A156N was selected by the reason of the homologous residue D ionic-binding with Ca2+ and H-binding with carbohydrates in the majority of the C-type lectins. The substitution of nonpolar F159 located near the mannose residue in the oligomannan for the positively charged residue K could facilitate the additional formation of hydrogen bond between the MBL-*AJ* lectin and the model oligosaccharide.

**Table 2:** The model oligosaccharide interaction energy and binding affinity with MBL-*AJ*.

Mutant	Solvation,* kcal/mol	Affinity,** kcal/mol
Wt	−54,70	−7,69
W100A	−41,77	−5,66
Q103H	−46,35	−6,62
A156N	−58,68	−8,49
F159K	−65,58	−8,21
A156N/F159K	−74,53	−9,49

*- The molecular mechanics generalized Born interaction energy is the non-bonded interaction energy between the receptor and the ligand and comprises van der Waals, Coulomb and generalized Born implicit solvent interaction energies [Labute, 2008].

**- The estimated binding affinity is that of the GBVI/WSA dG scoring function reported in units of kcal/mol.

**Figure 12:**
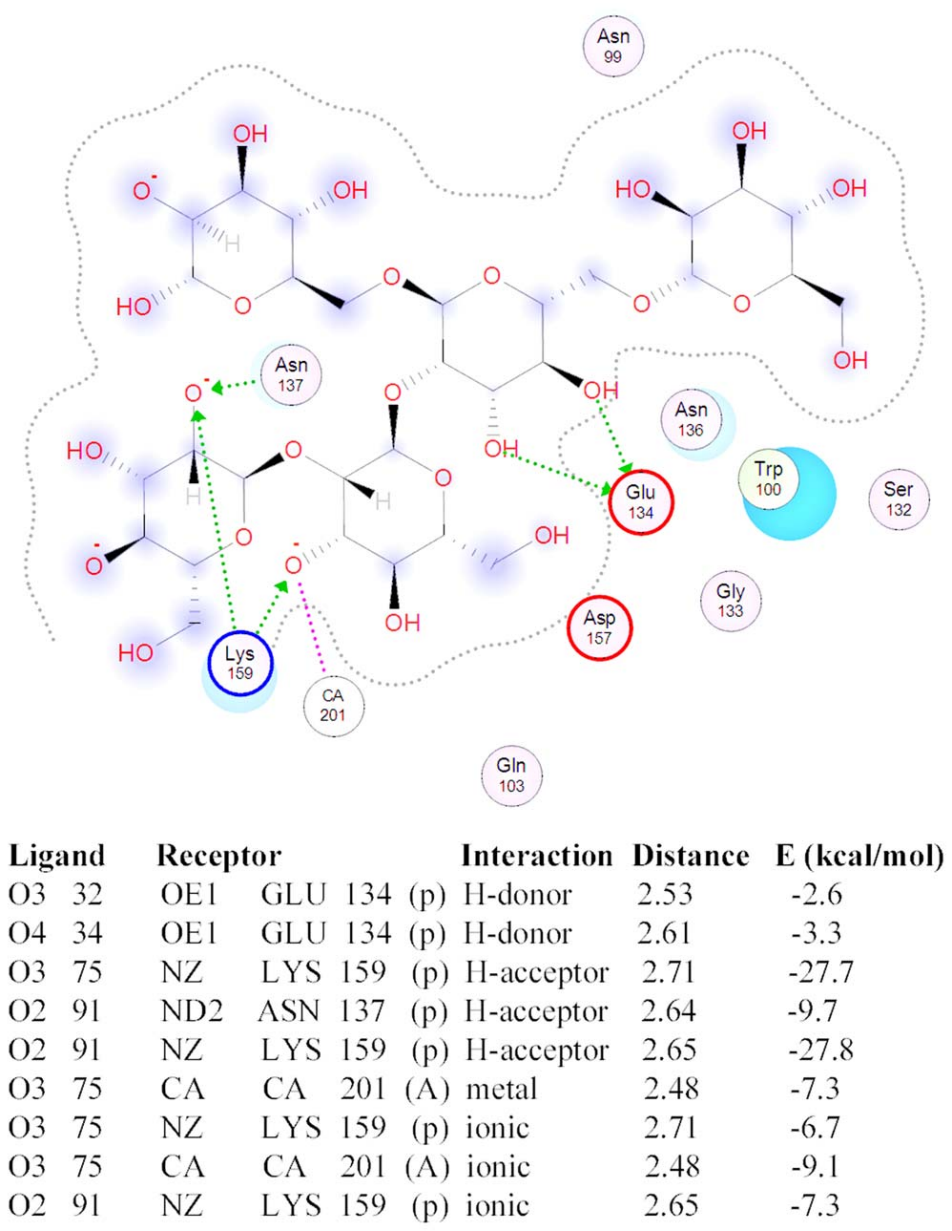
2D-Diagram of the oligosaccharide molecular contacts with A155N/F159K MBL-AJ mutant and contacts of model oligosaccharide with A155N/F159K MBL-AJ mutant.

Calculation revealed that the double mutants A156N/F159K exhibit a lower binding energy and higher affinity than the wild-type lectin (Table 2). Replacing enhances the interaction of the oligosaccharide with the lectin by additional energetically more favorable hydrogen and ionic bonds (Fig. 12).

In the case of the A156N, F159K mutants, their binding energies are lower than the wild-type lectin's one, but higher than the energy of the double mutant A156N/F159K. Mutations W100A and Q103H decrease lectin binding affinity to the carbohydrate moiety. Thus, *in silico* it was demonstrated that the specificity and high affinity of the MBL-*AJ* lectin are determined by its interaction with the monosaccharides from the main and branching chains of the model oligosaccharide.

**Figure 13:**
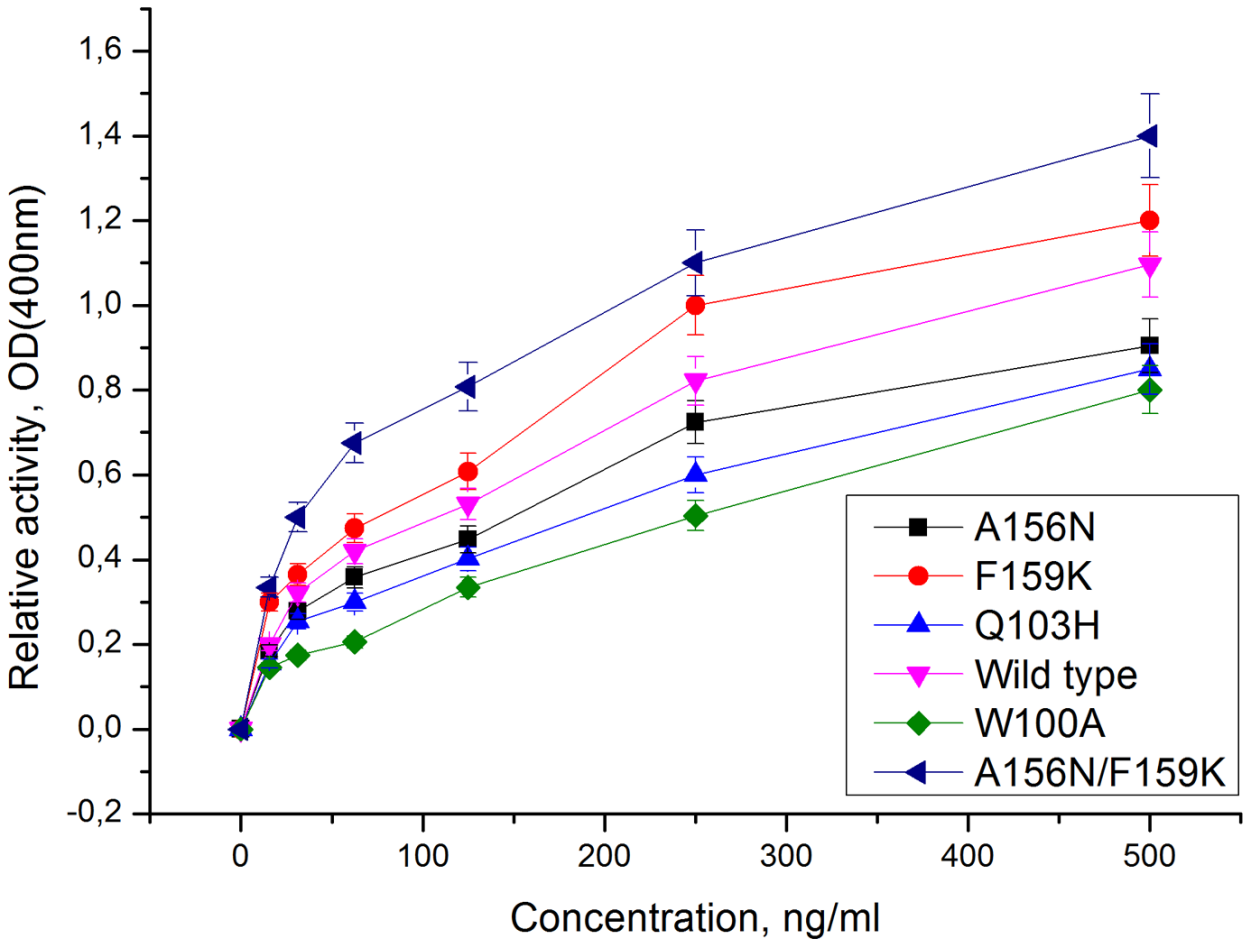
Lectin-binding activity of the bifunctional hybrid *Cm*AP/MBL-*AJ* mutants. The lectin-bound complexes with mucin (axis X) were monitored by measuring the phosphatase activity of *Cm*AP/MBL-*AJ* (axis Y).

For the experimental testing of the lectin-binding activity, W100A, H103Q, A156N, F159K and A156N/F159K mutants were expressed in *E.coli*. The chimeric mutants of *Cm*AP/MBL-*AJ* remained the lectin activity in the range of the increasing of the level of affinity W100A– H103Q– A156N– F159K to mucin that was in accord with the calculated data (Table 2, Fig. 13). The W100A, Q103H, A156N mutations decreased the lectin-binding activity in comparison with the wild-type *Cm*AP/MBL-*AJ* by 14±3%, 12±2% and 8±2%, respectively.

The substitution F159K enhanced the lectin binding of *Cm*AP/MBL-*AJ* by 10±3% due to the formation of the additional hydrogen bond with the OH-group of the mannose residue of the substrate (Fig. 12, 13). Surprisingly, the double A137N/F159K mutation had cumulative positive effect on the lectin affinity of *Cm*AP/MBL-*AJ*, increasing its activity by 25±5%, 40±2% and 28±3% in comparison with the wild-type *Cm*AP/MBL-*AJ* and single A137N and F159K mutants, respectively (Fig. 13).

## Discussion

Currently, construction of the fusion or tagged proteins is a useful method to obtain chimeric molecules with an improved functionality or, moreover, dual mode of action such as enzymes with specific binding activities [25], [26], [27], [28]. Here we report on genetic engineering and overexpression in *E.coli* of the bifunctional hybrid protein *Cm*AP/MBL-*AJ* with the alkaline phosphatase *Cm*AP of marine bacterium and the Far Eastern holothurian lectin MBL-*AJ* activities in the purpose of further improving the enzyme-linked lectin assay (ELLA) for diagnosing of cervical cancer [5], [13], [29]. Although the holothurian MBL-*AJ* and mammalian lectins, including human MBL, had common properties to recognize bacterial mannans, the differences in the carbohydrate-binding specificities were found to be significant [5]. It has been previously shown that holothurian lectin MBL-*AJ* interacts with the promising cancer biomarkers in the following order of the lectin-binding affinity: CEA, embryonic α-1-acid glycoprotein, trophoblast-specific β1-glycoprotein and α-fetoprotein isolated from the abortive and retroplacental blood [13], [30], [31], [32]. MBL-*AJ* is not inhibited by monosaccharide, which is in the content of the normal human glycoconjugate's carbohydrate chains, and does not interact with the human blood serum components. On the base of the unique carbohydrate-binding domain that defines the MBL-*AJ* ability to distinguish microheterogeneity of the malignant or normal cell glycoconjugates, a novel method of cervical cancer diagnosis has been developed [13]. The method was adapted to determine the quantitative level of the lectin-binding forms of CEA in the vaginal secretions of patients. The vaginal secretions were collected from the proximal uterine cervix surface, irrespectively on the age and physiological state of patients. The method with the use of MBL-*AJ* has allowed identifying statistically reliable differences between the levels of lectin-binding CEA between healthy women and patients with cervical cancer, and between patients with benign and malignant neoplasms [13]. Moreover, it is important that MBL-*AJ*-based method give a possibility to assess tumours noninvasively. Loss of expression of the MBL-*AJ* ligands was found in cervical cancer. According to the results, the concentration of the MBL-*AJ*-binding CEA of the healthy patients and the patients with the benign neoplasm were determined to be 48.5±11.8 U/ml versus the concentration of 11.4±7.5 U/ml of the patients with the cervical cancer diagnosis. The total CEA, which can be synthesized by both malignant and normal cells, were excluded from analysis because the cervical specimens were collected from the local source of the cancer CEA biosynthesis. A high sensitivity and specificity of the MBL-*AJ*-CEA interaction allowed detecting the lectin-binding structures in the vaginal secretion in the concentration of CEA 3–50 ng/mL [13]. The method specificity and sensitivity were 93.6 and 87.8%, respectively, for patients with cervical cancer with the cut-off level of 12.74 ng/mL. Whereas the method prognostic valuation was calculated to be 87% and 95.2% for the positive and negative diagnosis, respectively. Thus, this method has advantages compared to those associated with determining of concentration of CEA and squamous cell carcinoma antigen (SCC) in blood serum [2], [13], [14].

However, the wild-type lectin MBL-*AJ* derivation from the holothurian *A. japonicus* coelomic liquid has many restrictions, namely: low concentration of MBL-*AJ* in the native source, preservation of the wild life of the Far Eastern holothurian *A. japonicus*, the narrow habitat of the endemic species of the holothurian. A recombinant analogue of MBL-*AJ* was attempted to be produced in the *E. coli* Top10/pQE_80L expression system [29]. However, the protein was expressed in the body inclusion, and its refolding resulted in 20%-yield of the soluble recombinant lectin of 69% of homology with the wild-type MBL-*AJ* by the level of interaction with the antibodies against MBL-*AJ*. The recombinant production of the bifunctional hybrid *Cm*AP/MBL-*AJ* reached the full solubility and functionality of the lectin MBL-*AJ*. Moreover, the alkaline phosphatase *Cm*AP fused with the lectin MBL-*AJ* by the long flexible linker facilitated the recombinant protein purification monitoring as well as the lectin binding assay, eliminating the additional steps, namely: production of the antibodies against MBL-*AJ*, labeling the MBL-*AJ* antibodies by horsh peroxidase or alkaline phosphatase and incubation of the labeled MBL-*AJ* antibodies with the ligand-binding lectin MBL-*AJ*.

Although the alkaline phosphatase *Cm*AP included in the hybrid *Cm*AP/MBL-*AJ* has N-end and C-end fusion proteins, its activity is not limited. The N-ends of both lectin subunits in *Cm*AP/MBL-*AJ* diverge angularly in the reverse direction from each other, permitting the AP modules of the dimeric hybrid lectin to function independently (Fig. 10). However, the tetramerization of *Cm*AP/MBL-*AJ* occurs, probably, by the other mechanism than in the wild-type MBL-*AJ*. The first N-end cysteins of the dimeric MBL-*AJ* subunits are blocked by linker (G_4_S)_3_ connecting to the *Cm*AP module, whereas they form else two disulfide bonds in the native homotetrameric MBL-*AJ* (Fig. 7, 8, and 9). It has been suggested that tetramer of the bifunctional hybrid *Cm*AP/MBL- *AJ* is formed by the only hydrophobic interactions between the dimers of the lectin parts of *Cm*AP/MBL-*AJ* at the highly alkaline pH≥9 (data not shown).

The applications for molecules that bind proteins keep expanding, and issues of affinity, specificity, robustness, and time for development are still in focus. The potential limitations of using chimeric fusion molecules in clinical medicine include induction of antibodies to novel biological agents, and this will need to be specifically investigated. However, high enzymatic activity and stability of *Cm*AP/MBL-*AJ* in different buffers and high affinity and specificity of its lectin part ensure good efficacy and minimal side effects of reliable detection of different carbohydrate structures in the *Cm*AP/MBL-*AJ*-captured glycoproteins in diagnostic applications.
